# Intensity output and effectiveness of light curing units in dental offices

**DOI:** 10.4317/jced.54756

**Published:** 2018-06-01

**Authors:** Baharan-Ranjbar Omidi, Armin Gosili, Mona Jaber-Ansari, Ailin Mahdkhah

**Affiliations:** 1 Assistant Professor, Department of Operative Dentistry, Faculty of dentistry, Qazvin University of Medical science, Qazvin, Iran; 2 Assistant Professor, Department of Orthodontics, Faculty of dentistry, Golestan University of Medical science, Gorgan, Iran; 3 Dentist, Private Practice, Tehran, Iran; 4 Post-graduate Student of Operative Dentistry, Faculty of Dentistry, Qazvin University of Medical Science, Qazvin, Iran

## Abstract

**Background:**

The aims of the study were measuring the light intensity of light curing units used in Qazvin’s dental offices, determining the relationship between the clinical age of these units and their light intensity, and identifying the reasons for repairing them.

**Material and Methods:**

In this cross-sectional study, the output intensity of 95 light curing devices was evaluated using a radiometer. The average output intensity was divided up into four categories (less than 200, 200-299, 300-500, and more than 500 mW/cm2). In addition, a questionnaire was designed to obtain information mainly about the type, clinical age, and frequency of maintenance of the units and the reasons for fixing them. Data were analyzed using Kolmogorov-Smirnov, chi-squared, and t-tests (*p*< 0.05) on SPSS 24.

**Results:**

A total of 95 light curing units were examined, with 61 (64.2%) of them being of the LED type and 34 (35.8%) of the QTH type. While average light intensity in LED units was significantly higher than in QTH devices, the two device types were not significantly different regarding desirable light intensity (i.e., ≥ 300 mw/cm2). A negative correlation was observed between clinical age and light intensity. In addition, bulb replacement in QTH devices was over three times as much as in LED units. Also, repairing QTHs was more than twice as much frequent as fixing LEDs. The most common reason for repair was the breakage of the tip of the device.

**Conclusions:**

The light intensity of LED units is significantly higher than that of QTH devices, and the frequency of repairing in QTHs was significantly more than in LEDs. Furthermore, light intensity decreases with aging, and dentists should regularly monitor the conditions of light units.

** Key words:**Light curing unit, radiometer, light intensity, dental equipment, dental offices.

## Introduction

Over three decades have passed since the beginning of the extensive use of composite resins in dentistry, and the demand for using esthetic restorative materials is still on the increase (1). Resins must begin polymerization in order to perform operation. During this process, monomer units bond with each other to build long and heavy polymers. Due to the increased use of optical composites, the importance of polymerization has become more prominent. The strength of these restorations depends on the degree of polymerization of composite resins. Incomplete polymerization produces adverse biological effects, increasing water absorption, composite solubility, and reducing hardness. Various factors contribute to the polymerization of the composites, and they include the wavelength and intensity of the output of light curing units, duration of radiation, dimensions and location of the dental cavity, direction and distance of the tip of the device (related to the composite), the composition of the composite, the wavelength and bandwidth of the curing light, the intensity of the curing light, the irradiation time, and color and thickness of the composite (2,3). In composite resins, camphorquinone is the light-sensitive component, which responds to irradiation by creating free radicals and initiates the polymerization process (4). An appropriate intensity of light with the maximum absorption wavelength range of camphorquinone is the main factor in the polymerization of these resins. If the light output intensity decreases, it will adversely influence the clinical and cosmetic performance. The light intensity of curing devices is defined by the International Organization for Standardization as the ISO 4049 standard, which recommends an intensity of 300 mW/cm2 with a wavelength bandwidth of 400-515 nm on the tip of the light curing device. At this standard wavelength, the minimum depth of cure is assumed to be 1.5 mm, which is 50% of the length of the composite specimen (3). The reduction in the light intensity of the device can affect the success rate of the restorative methods via reducing the degree of convergence of composites, which leads to an increase in microleakage and recurrent caries (5).

The light source for polymerization of composite resins are available in four types: quartz-tungsten-halogen (QTH), light-emitting diode (LED), plasma arc curing (PAC), and argon laser. Halogen-based curing lamps have several limitations. One of the main disadvantages of these lamps is the high energy consumption. Only 1% of the consumed energy by these devices turns into light and almost all the remaining energy is converted into heat. The heat generated by these lamps should be eliminated, and this requires expensive thermal filters. Cooling fans are also loud and bulky. Also, the longevity of halogen lamps is short (between 40 and 100 hours) (6). In 1995, Mills and colleagues presented solid-state LED technology for the polymerization of dental materials capable of being activated with light. In LEDs, instead of hot strands as used in halogen lamps, semiconductor connections are employed to produce light. These lamps have a very long shelf life of about 1,000 hours and can withstand mechanical shocks and vibrations with very low error rates (7). LEDs are also capable of producing blue light at a wavelength of 440-480 nm. LEDs can be cordless and are almost silent while being operated (7).

In QTH and LED light curing devices, the main factors affecting the intensity of light output are: inappropriate performance of the lamp and filter, breakage and pollution of the device tip, the blurring of the bulb, the failure of electrical components, and defect in light transmitting fibers (6,7). In these devices, if maintenance is not carried out routinely, after a while, there will be some problems with the lamp, fan, or power supply (8).

There are two main problems with the quality of cured resin composite in the office:

1) Composite surface hardness is not a reliable guide because even at a low light intensity, the surface can sufficiently harden while the depth of the cure is not adequate. Moreover, it is impossible for the dentist to distinguish completely-cured composite resin from the one incompletely cured using a device with a low light intensity (9).

2) The output light of the device decreases as the device is used more, but this is not detectable by the unarmed eye because sometimes a seemingly bright light is not suitable for wavelengths. Furthermore, insufficient radiation intensity is not always compensated for by prolonging exposure time (10). Therefore, a digital radiometer is needed to measure the intensity of the curing light of the units to determine when the device needs to repaired or replaced (11).

The aims of the present study were four-fold: 1) measuring the light intensity of light curing units used in the offices, 2) comparing the light intensity of LED and QTH units, 3) determining the relationship between the clinical age of these devices and their light intensity, and 4) exploring the reasons for and the frequency of repairing these units.

The findings of this study underscore the importance of timely fixing or replacing defective light curing devices, which can consequently ensure the continued quality of restorative treatments. This improvement can increase public health in the long run. It is believed that these findings can be used in the macro-planning of the health system.

## Material and Methods

The following criteria were used to include private dental offices in this study: 1) the person operating the office had to be a general dentist rather than a specialist, and 2) the dentist had to routinely use composite resins for tooth restoration. The 2016 alphabetical listing of dentists published by the Qazvin University of Medical Sciences was used to identify dental offices located in Qazvin. All the 105 offices on the list were contacted via telephone to determine if they satisfied the criteria. Of these offices, 88 fulfilled both criteria. The dentists in charge of these latter offices were contacted by telephone to explain the rationale and methodology of the study and to obtain his or her consent. Ultimately, 70 dental offices agreed to participate. Upon this, an appointment was arranged for a visit to the office.

To measure light intensity, an analog radiometer (DigiRate, Monitex, Taiwan) with a range of 0 to 1,000 mW/cm2 was used. The radiometer was sent to the Laboratory of Optics at Sharif University of Technology in Tehran, Iran to confirm its performance. Once approval was received, the offices were visited to perform the measurements. At each office, a few minutes were spared to allow the radiometer to match up with the ambient temperature. Then, three measurements of light intensity were recorded for each light curing unit, and the average was reported as the final measure. At the intervals between visits, the accuracy of the radiometer was checked against a radiometer at the restorative dentistry laboratory of the Qazvin University of Medical Sciences. Light intensity of less than 300 mW/cm2 was considered unacceptable (3), and a device with a light intensity of less than 200 mW/cm2 was regarded unusable (12).

In addition, a questionnaire, designed by the researchers, was used to obtain information about type of light curing unit, the age of the unit, the frequency of maintenance, the reasons for repair, the date of the last repair, the number of times the bulb had been replaced and the last time this had been done, the typical duration of light irradiation at each restoration, the availability of a radiometer in the office, and the number of office hours per week.

Once the data had been collected, they were analyzed using SPSS 24 (IBM Corporation, USA, 2016). The mean and standard deviation were used to determine the mean light intensity of the light curing units. The Kolmogorov-Smirnov statistic was used to determine the normality of the distribution of the light intensity scores.

The clinical age of the device was calculated using the following equation:

Clinical age = time in use (in terms of year) × 52 (number of weeks in a year) × number of working days of the office per week × average number of instances of the use of the device per day × average duration of light exposure (in terms of second) at each instance of the use of the device.

Pearson product-moment (r) correlation coefficient was used to determine the correlation between the clinical age and the light intensity of the device.

Independent-samples t-test was used to compare the light intensity of LED and QTH units.

The chi-squared test was employed to find out about the correlation between device type on the one hand and the frequency of bulb replacement, the frequency of device repair, and the reason for device repair on the other hand.

Statistical significance for all tests was set at an α level of < 0.05 (2-tailed).

-Ethical considerations

The study was endorsed by the Research Ethics Committee of Qazvin University of Medical Sciences under the approval ID of IR.QUMS.REC.1394.642. Additionally, the information contained in this study does not mention the names of the honorable participating dentists.

## Results

-Device type

A total of 95 light curing units were examined: 61 (64.2%) were LED and 34 (35.8%) were QTH.

-Correlation between device type and light intensity 

The light intensity of LED and QTH units is shown in  Table 1. As can be seen, about 93% of LED devices had a desirable light intensity (i.e., ≥ 300 mw/cm2), whereas this figure is around 97% for QTH units. Results from the t-test showed that the relationship between the two variables was insignificant regarding desirable light intensity. However, average light intensity in LED units was significantly higher than in QTH devices.

**Table 1:**
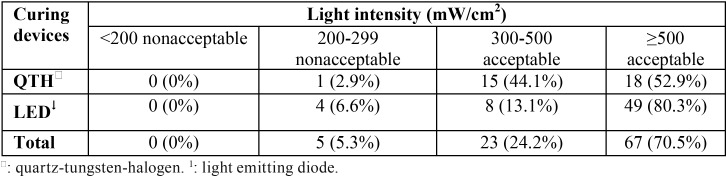
Distribution of acceptable/nonacceptable light intensity for the curing devices under study.

-Relationship between clinical age and light intensity

Pearson r revealed a negative relationship between clinical age and light intensity ( Table 2).

**Table 2:**
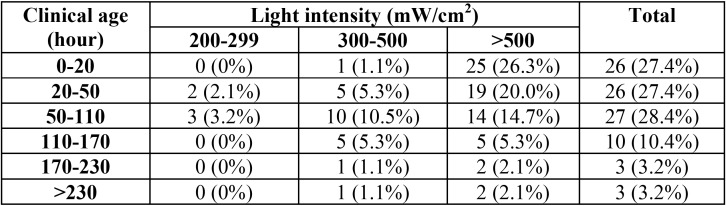
Clinical age and light intensity of light curing devices.

-Relationship between device type and the frequency of replacing device bulbs

 Table 3 shows the relationship between device type and the frequency of replacing the bulb. It is evident that bulb replacement in QTH devices was over three times as much as in LED units. The chi-squared test showed a significant relationship between the two variables.

**Table 3:**
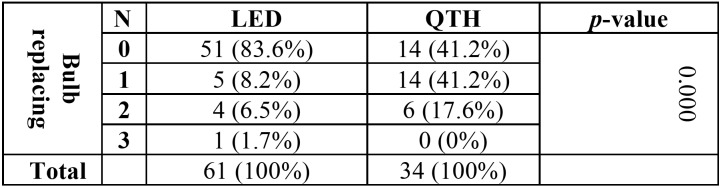
Frequency of replacing the bulb in LED and QTH devices.

-Relationship between device type and the frequency of device repair 

The relationship between device type and the frequency of device repair is given in  Table 4. According to the chi-squared test results ( Table 4), the relationship between these two variables was statistically insignificant.

**Table 4:**
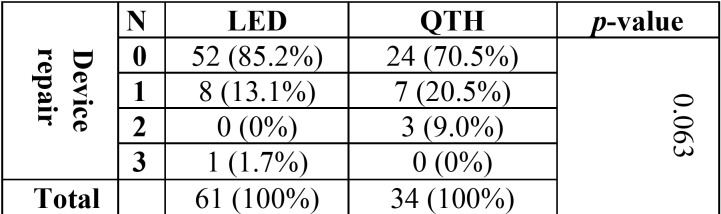
Relationship between device type and the frequency of device repair.

-Relationship between device type and the reasons for device repair

According to  Table 5, the most common reason for repair was the breakage of the tip of the device. More specifically, this was the cause in 78% of all instances of repair in the case of LED devices, and 40% in the case of QTH units. The chi-squared test revealed a significant relationship between the two variables.

**Table 5:**
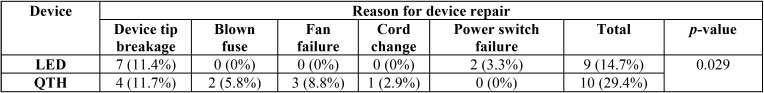
Relationship between device type and the reasons for device repair.

The devices studied are based on the model presented in  Table 6. The most common model used in dentistry offices in Qazvin is the Woodpecker model, which has a good radiation intensity in all cases. It was also found that the Gnatus light cure device had the lowest percentage of light-intensity.

**Table 6:**
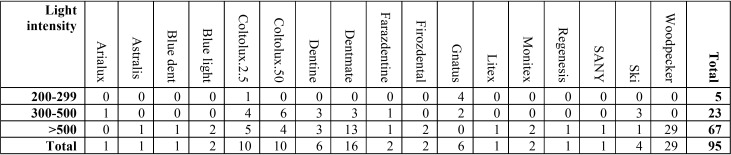
Models of light curing units used in Qazvin’s dental offices (2017).

## Discussion

This study found that most of the devices were LED-type. Despite the popularity of halogen devices in the past, there are some problems with QTHs. As noted above, these include the short life span of halogen lamps and the fact that the reflector and filter can undergo a reduction in efficiency over time. Overheating is one of the other disadvantages of such devices (6). However, these issues are less frequently seen in LEDs, which has resulted in a rapid growth of the use of these units. Another upside of LEDs is that their light output wavelength peak of 456 nm matches the absorption peak of camphorquinone (13). However, initiators other than camphorquinone have slightly different absorption spectra. Thus, it is better to use halogen devices with these materials, but they may not fully cured with LEDs because the light output wavelength range of halogen is wider than that of LEDs. On the other hand, spectral purity in LED devices allows better polymerization of composites with camphorquinone. In addition, LEDs do not emit too much heat (14).

The first aim of the present study was measuring the light intensity of light curing units in use in the dental offices of the city of Qazvin. It was found that none of the devices had a light intensity of less than 200 mW/cm2 and that 6.6% of LEDs, 2.9% of QTHs, and 5.3% of all total devices had a light intensity of less than 300 mW/cm2, the desirable level. Unlike this study, Miyazaki *et al.* (10), Barghi *et al.* (1994), (6) and Martin (15) reported high percentages: 41.9%, 29.7% and 27%, respectively.

In 2009, Javaheri and Ashreghi (16) concluded that the light intensity of 27.4% of the devices in their study was less than desirable. Since that study was also carried out in Qazvin, the much lower percentage reported in the present study can be attributed to the increased awareness among the dentists of the necessity of continued maintenance of light curing units over recent years. On the other hand, our findings are similar to those of Barghi *et al.*, (11) and Savadi Oskoee (8), who reported an undesirable percentage of 10.4% and 10%, respectively.

Overall, the wide variety in the results reported in the literature can be ascribed to the variety in the devices in terms of type, maintenance, and clinical age. Indeed, the clinical age of 94% of devices in the present study did not exceed 170 hours. Another possible cause is the cleanliness of the tip of the devices as research has shown that the gradual buildup of the debris of composite resins on the curing tip can significantly decrease light intensity (12,17). This was not measured in the present study.

Another observation was that the light intensity of LEDs is significantly higher than that of QTHs, which may be due to the difference in the output light spectrum and clinical age of devices. This finding is consistent with a study by Hegde *et al.* (18).

It was also observed that the clinical age of the devices is negatively correlated with the intensity of light. A similar finding was reported by Barghi *et al.* (6), Martin (15), Poulos and Styner (19) and Friedman (20). However, Javaheri and Ashreghi (16) concluded that there is no significant correlation between clinical age and light intensity. Two possible reasons for the difference in the results can be the use of different models of light curing devices in different studies and different levels of device consciousness on the part of participating dentists.

Furthermore, a significant relationship was found between the frequency of bulb replacement and the type of device, with greater frequency in the case of QTHs (58.8% as opposed to 16.4% for LEDs). This is attributable to the longer life span of an LED lamp, which is over 1,000 hours as opposed to 100 hours for a QTH lamp (7).

Also, the relationship between the frequency of lamp replacement and light intensity did not teach significance. However, it should be noted that only a small number of devices in our study had their lamp replaced. It is worth noting at this point that more frequent bulb replacement is an indicator of the aging of the device, which in turn signals the aging of the other parts of the device as the potential factors contributing to light intensity. The study also found that the main reason for repair was the breakage of the tip of device. This clearly shows the vulnerability of the tip to breakage.

Finally, the most common model used in the dental offices of Qazvin was Woodpecker (Woodpecker Med. Instrument, Guilin, china), which was found to have the favorable light intensity of all models. It was also found that the Gnatus model (Gnatus Medical-Dental Equipments, Brazil) has the lowest light intensity, but it should be noted that there were only a small number of Gnatus devices, which were also very old. However, the dentist’s attention to the periodic measurement of the intensity of the device and doing the necessary repairs can increase the efficiency of the device.

## Conclusions

This study concluded the following:

The light intensity of the light curing devices in the dental offices of Qazvin was acceptable (i.e., over 300 mW/cm2).

1. The light intensity of LEDs was significantly higher than that of QTH devices.

2. Light intensity decreases with the aging of the device.

3. The frequency of bulb replacement in QTHs was significantly higher than in LEDs.

4. There was a significant relationship between device model and light intensity.

5. For dentists who do not carry out a regular maintenance of light curing devices, it is better to use LEDs because these devices have a higher light intensity and a longer bulb life span.

6. Regarding the fact that reduction in light intensity can affect the success rate of restorative methods, light intensity measurement should be carried out regularly.
